# Etymologia: **Cyprinid Herpesvirus**

**DOI:** 10.3201/eid1612.ET1612

**Published:** 2010-12

**Authors:** Carol Snarey

**Affiliations:** Author affiliation: Centers for Disease Control and Prevention, Atlanta, GA, USA

**Keywords:** Etymologia, Cyprinid Herpesvirus, cyprinids, carp

## [sip′ri nid] [hur′pēz vi′rəs]

Cyprinids, members of the large freshwater fish family *Cyprinidae*, take their name from the Greek *Kypris*, also another name for the Aphrodite, Greek goddess of love and beauty. It refers to the island of Cyprus, alleged to be the site of her birth. The term herpesvirus derives from Greek *herpes*, a spreading eruption, and the Latin word for poison. This virus is an emerging infection in common carp (*Cyprinus carpio carpio*) and koi (*C. carpio koi*).

**Sources:** Dorland’s illustrated medical dictionary, 31st ed. Philadelphia: Saunders Elsevier; 2007; www.statemaster.com/encccyclopedia/Cyprinids.

